# Structure and Dynamics of the Magnetite(001)/Water Interface from Molecular Dynamics Simulations Based on a Neural Network Potential

**DOI:** 10.1021/acs.jctc.4c01507

**Published:** 2025-02-13

**Authors:** Salvatore Romano, Pablo Montero de Hijes, Matthias Meier, Georg Kresse, Cesare Franchini, Christoph Dellago

**Affiliations:** † Faculty of Physics, University of Vienna, Kolingasse 14-16, 1090 Vienna, Austria; ‡ Vienna Doctoral School in Physics, University of Vienna, Boltzmanngasse 5, 1090 Vienna, Austria; § VASP Software GmbH, Berggasse 21, A-1090 Vienna, Austria; ∥ Department of Physics and Astronomy “Augusto Righi”, Alma Mater Studiorum - Università di Bologna, Bologna 40127 Italy

## Abstract

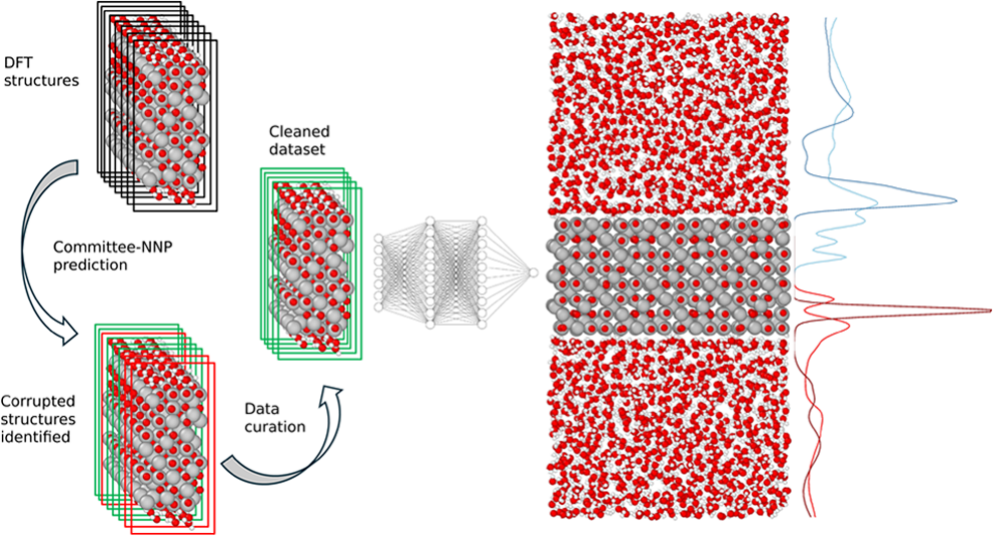

The magnetite/water interface is commonly found in nature and plays a crucial role in various technological applications. However, our understanding of its structural and dynamical properties at the molecular scale remains still limited. In this study, we developed an efficient Behler-Parrinello neural network potential (NNP) for the magnetite/water system, paying particular attention to the accurate generation of reference data with density functional theory. Using this NNP, we performed extensive molecular dynamics simulations of the magnetite (001) surface across a wide range of water coverages, from single molecules to bulk water. Our simulations revealed several new ground states of low coverage water on the Subsurface Cation Vacancy (SCV) model and yielded a density profile of water at the surface that exhibits marked layering. By calculating mean square displacements, we obtained quantitative information on the diffusion of water molecules on the SCV for different coverages, revealing significant anisotropy. Additionally, our simulations provided qualitative insights into the dissociation mechanisms of water molecules at the surface.

## 1 Introduction

Iron oxide interfaces are common in geochemistry and biology and they play crucial roles in phenomena such as corrosion and nucleation.^1−6^ Various technological areas rely on iron oxide interfaces, particularly in applications related to energy conversion and storage, nanoparticles, spintronics, and even biomedicine.^7−10^ The importance of these interfaces and the need for a deeper understanding have driven much experimental and computational research on this topic (see ref (11) for an extensive review on iron oxides). The interface between magnetite (Fe_3_O_4_) and water is a prominent example of iron oxide interfaces.^4,12−18^ The bare (001) and (111) surfaces of magnetite have been found to be the most stable ones^19,20^ and, more recently, Bliem et al.^21^ discovered that the (001) surface undergoes a reconstruction known as the Subsurface Cation Vacancy (SCV) model (see Figure 2), which involves the three outermost layers of magnetite. Later, water agglomeration on the SCV has been studied experimentally and computationally via Density Functional Theory (DFT) by Meier et al.^22^ Their study showed that energetically favorable configurations for various water coverages on the SCV (up to 8 H_2_O/u.c.) are stabilized by the presence of a partially dissociated water molecule. While such DFT calculations can provide insights on atomistic structures at the interface,^23−26^ they are computationally too expensive to capture spatially correlated thermal fluctuations and dynamics occurring on extended time scales.

In this study, we have developed a Neural Network Potential (NNP) following the methodology proposed by Behler and Parrinello,^27−29^ enabling efficient simulations with *ab initio* accuracy of the interface between the SCV of magnetite and water. The reference data used to train the potential were obtained from *ab initio* calculations based on DFT following a multistep procedure, which started with DFT-based molecular dynamics (MD) simulations enhanced by kernel-regression via “on-the-fly” learning.^30,31^ A committee of NNPs^32^ trained on data generated at this stage was then used to complete the exploration of the configurational space, iteratively expanding the data set. Here, structures exhibiting large force disagreements were retained, calculated *ab initio*, and added to the data set, which was used to train the subsequent generation of the committee of NNPs. These steps constitute one “committee iteration”. During data acquisition, special care was taken to address convergence problems of the DFT calculations related to the magnetism of Fe_3_O_4_. To deal with these complications, we have devised a protocol that ensured that such “corrupted” data were not part of the reference data set.

We employed the newly developed NNP to carry out extensive molecular dynamics simulations of water molecules interacting with the SCV, from low coverages to liquid water. First of all, we addressed the dynamics of the stable water trimers (with linear and triangular shapes) that are energetically equivalent at the DFT level.^22^ The linear trimer, which experimental observations identified as the predominant configuration, was found to be more than 30 times as likely to occur as the triangular trimer. The NNP proved to be an effective tool for the systematic search of energy minima, and our simulations revealed new ground states of low water coverages on the SCV. The NNP simulations enabled the detailed study of the water dissociation mechanism and the calculation of the two-dimensional mean square displacements (MSD). We extracted quantitative data on the diffusion dynamics of water molecules at the SCV surfaces, revealing a strong dependence on the coverage and significant directional anisotropy. For the liquid water case, the density profile of water at the magnetite interface exhibited distinct layering, with bulk-like behavior emerging only beyond a distance of approximately 15 Å.

The remainder of this article is organized as follows. In Section 2 we line out the methodology used in our work, paying particular attention to data acquisition and curation. The results obtained for various water coverages are presented in Section 3. A discussion and conclusions are provided in Section 4.

## 2 Methods

In this section, we review the methodology used in our work. In particular, we describe how the data set for the training of the NNP was constructed and cleaned of DFT inconsistencies.

### 2.1 Data Acquisition

#### 2.1.1 Preliminary Data Set

In the initial phase of data acquisition, DFT-MD simulations were carried out with the Vienna *ab initio* Simulation Package (VASP)^33^ using the projector-augmented wave (PAW) formalism.^34^ Following Meier et al.,^22^ we chose the vdW density functional optB88-DF+*U*, with the effective on-site Coulomb repulsion term *U*_eff_ = 3.61 eV added to describe the metal oxide system. The PAW potentials H_GW_, O_GW_, and Fe_pv_ were used. Based on the nonlocal van der Waals density functional of Dion et al.,^35^ the optB88-vdW functional was proposed in ref (36) for the description of hydrogen-bonded complexes and it has been successfully applied before in single-point calculations of the magnetite-water interface.^22^ The plane wave energy cutoff was set to 500 eV and we used a Monkhorst–Pack mesh to sample the Brillouin zone with (2 2 1) subdivisions along the reciprocal lattice vectors. The electronic self-consistency loop was stopped when the change in energy was smaller than 1 × 10^–5^ eV. The magnetite hosts octahedrally and tetrahedrally coordinated iron atoms, which we will refer to as octahedral and tetrahedral irons. The initial guess of the local magnetization of cations was +4 μ_B_ for all octahedral irons and −4 μ_B_ for tetrahedral irons. In the bulk, the minimization led to some octahedral irons having magnetizations between +3.6 and +3.8 μ_B_, corresponding to a 2+ oxidation state, whereas the magnetizations of the other irons remained close to these initial values.

At this initial stage, to improve configurational sampling we employed the “on-the-fly” learning protocol with a kernel-based model implemented in VASP.^30^ For the machine learning potential, a radial cutoff of 8 and 5 Å for the two-body and three-body terms, respectively, and 8 basis functions were employed for the pair descriptors and for the three-body descriptors. The maximum angular quantum number was set to *L*_max_ = 4. To speed up sampling, a large time step of 1.5 fs was chosen, which we compensated with an increased mass of 8 amu for the hydrogen atoms.

The building block of our initial configurations was a magnetite slab with 64 oxygen and 44 iron atoms exposing two SCV surface unit cells in the (001) direction (hereafter we write u.c. to refer to the surface unit cell) due to periodic boundary conditions (PBC) applied in all directions. At each of the two SCVs, water was arranged both randomly (with varying coverages from 0 to 12 H_2_O/u.c.) and as the minimum energy configurations proposed by Meier et al.^22^ From these setups, temperature ramps from 160 to 350 K were performed via the DFT-MD accelerated by on-the-fly machine learning. This protocol produced our first reference data made of approximately 2000 independent structures harvested from trajectories with a total length of 55 ps. With this data set, we trained a first committee of NNPs, which was then used in the second data acquisition stage.

#### 2.1.2 Committee of NNPs

We used n2p2^37^ interfaced with LAMMPS^38−40^ to train and run the committee of NNPs. We adopted the exponential atomic centered symmetry functions^41^ already developed for bulk water^42^ and expanded it to account for the bulk magnetite and the interfacial regions.^43^ We used a cutoff function of the cubic hyperbolic tangent form, which aids in preventing discontinuities in the forces,^28^ and the cutoff radius was set to 6.35 Å. Long-range interaction beyond twice the cutoff distance are not represented by the NNP. The parameters of the NNP were optimized with the multistream Kalman filter to minimize the cost function, which was constructed from the mean square deviation of the predicted energies and forces from their reference values.^37^

The first generations of NNPs were unstable, leading us to start with only *N* = 3 committee members and a relatively high disagreement threshold of 1 eV/Å for the forces. During the progress of the committee iterations, as the data set increased, different architectures were tested and the symmetry functions were refined by adding new ones and pruning after sensitivity analysis. The NNPs then improved, and we could expand the committee size to *N* = 8 members and lowered the threshold to 0.3 eV/Å to finer cover the configurational space.

As the iteration progressed, we systematically incorporated additional levels of water coverage into the data set, ultimately extending to a liquid slab comprising 68 water molecules in contact with the magnetite slab (forming two interfaces for the PBC). Bulk water structures with 128 molecules were also included.

In order to enable simulations of spontaneous water dissociation, an expected phenomenon on the magnetite surface, we ran simulations at higher temperatures (up to 500 K). At these temperatures, the dissociation rate was higher, providing more statistics on transition state configurations. Notably, this yielded structures with larger forces, which also contributed to the overall stability of the NNP. Eventually, we supplemented the data set with 342 *ab initio* calculations of ionized water structures (involving an OH^–^–H_3_O^+^ pair) to train the committee of NNPs such that it could handle scenarios involving OH^–^ also in water-like environments.

In total, around 15 committee iterations have been performed, resulting in the generation of 26,789 structures with *ab initio* energies and forces that constitute the final training data set. The validation set, containing 4807 structures, has been progressively constructed by randomly selecting structures from all the performed simulations.

As mentioned in the introduction, due to the DFT complications in covering the proper spin states of the magnetite, erroneous data were systematically introduced, and the standard committee protocol described here needed to be revised to include a cleaning step, which will be discussed in Section 2.2.

#### 2.1.3 Cleaned NNPs

**Figure 1:**
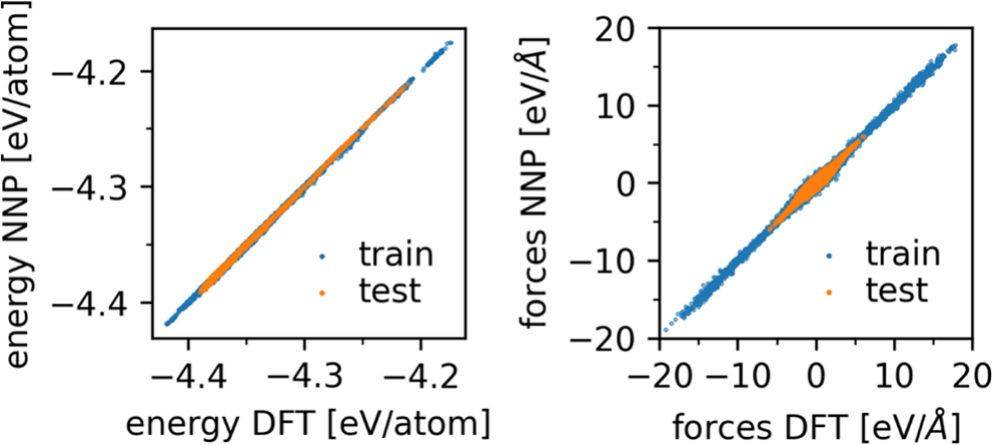
NNP predictions against the DFT reference for energies (left) and forces (right) for configurations in the training set (blue) and test set (orange).

The final committee comprises two distinct yet topological equivalent NNPs: one is meant to lead the production simulations while the other serves to validate the leader’s predictions. The final set of symmetry functions contains 156 radial and angular exponential symmetry functions, and the architecture is as follows: 2 hidden layers with 25 nodes per layer. The correlations of the leader NNP prediction and the DFT reference for energy and forces on the training and test set are shown in Figure 1. The NNP predictions have a root-mean-square error of 0.94 meV/atom in the energy and 110 meV/Å in the forces for the training set. This error is of the same order of magnitude as other Behler-Parrinello NNP not involving magnetism.^44−48^ On the validation set, we obtained better errors, namely, 0.91 meV/atom for the energy and 100 meV/Å for the forces.

### 2.2 Data Curation

*Ab initio* calculations of magnetic systems based on DFT are notoriously difficult and they often fail to converge or they do it to high energy configurations.^49^ In our calculations of the magnetite/water interface, we encountered several instances where, even after energy convergence, (i) the prediction of the magnetization was incorrect, with local magnetic moments seemingly randomly assigned, and (ii) convergence was to the correct magnetic state but with incorrect forces. While the exact cause of this instability in the calculation remains unclear, it may be due to the inherent complexity of the electronic energy in the presence of magnetism, where multiple local minima can be reached in the DFT energy self-consistency loop.^49^

#### 2.2.1 Data Curation through Consistency Checks

If these types of errors occur during *ab initio* MD runs, they pose a significant challenge, as they would compromise subsequent time steps and thereby invalidate the rest of the simulation. Moreover, if learning is done on the fly, also the ML force field is affected because corrupted structures and energies are introduced into the data set. To avoid these issues during the production of our preliminary data set, we devised a procedure to continuously monitor the total magnetization and temperature. Structures exhibiting incorrect total magnetization (accepted error ∼0.05%) or too high temperature (above 1200 K) were identified and removed from the training data set. If such a configuration was detected, the simulation was interrupted and restarted from the last valid configuration with velocities redrawn from a Maxwell–Boltzmann distribution. Moreover, the kernel-regression potential of the on-the-fly learning approach was passed on to the next MD attempt after being cleaned of the last corrupted data. Although this approach is impractical for conducting long *ab initio* MD simulations, it nonetheless proved to be effective in gathering sufficient data for training the first committee of NNPs.

#### 2.2.2 Data Curation Based on the NNP Committee

During the committee iterations, the DFT instability was less consequential, as energy calculations were performed only for single structures and the DFT calculations could be checked individually by inspecting the magnetization and the forces. Nonetheless, after this “manual” cleaning procedure, through the force prediction of the committee of NNPs, we spotted discernible outliers in the prediction vs reference plot. Remarkably, we observed that the force outliers belonged only to a small number of structures, and they were common outliers of all the NNP members. Therefore, we discovered that DFT calculations could converge in energy and magnetization with forces in the correct order of magnitude but still appear as outliers in the force prediction of the NNPs. Thus, if all the NNPs of the committee “agreed to disagree” on a particular structure, this was taken as an indication of corrupted data, and such structures were removed from the training data set. Eventually, the retrained committee of NNPs correctly predicted the energies and forces of all structures in the cleaned data set.

Although time-consuming due to manual intervention and retraining process, this systematic data cleaning procedure enabled the training of an ML force field with which accurate MD simulations could be carried out free from errors. These simulations would have been impossible with direct *ab initio* MD because of the instability of the electronic structure calculation.

### 2.3 Simulations Details and Analysis

#### 2.3.1 MD Details

**Figure 2:**
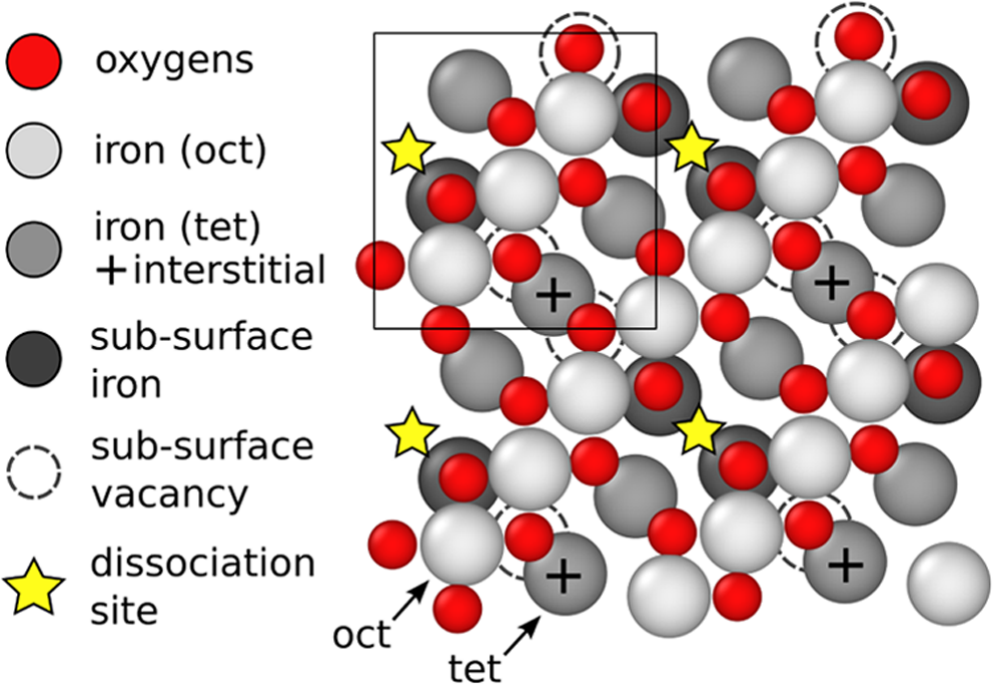
Top view of the SCV model. The surface unit cell  R45° with respect to the bulk) is enclosed in the square and replicated two times. The oxygen atoms are shown in red, and the iron atoms are in different shades of gray to distinguish between octahedral and tetrahedral irons (light and middle gray) and the subsurface octahedral irons (dark gray). The plus symbols indicate the interstitial tetrahedral irons that during the reconstruction are lifted from the subsurface layer, leaving there the subsurface vacancies (dashed circles). The active sites (stars) where the water dissociates one hydrogen (“dissociation sites”) are located in the tetrahedral iron rows in the spots free of irons.

The common setup of all of the initial structures of all of the MD simulations is a magnetite slab exposing two independent SCVs (see Figure 2). PBCs are applied in all directions: *x* and *y* with the periodicity of the SCV unit cell and z with a period always larger than the interaction range of the two interfaces. The unit cell of the magnetite slab contained 44 or 50 Fe (2 bulk layers of difference) and was symmetrically replicated in the *x* and *y* directions up to 6 × 6 (the specifications are shown in Table 1). The water coverages per unit cell vary for the low coverage regime from 1 to 8, i.e., up to the monolayer, and arrive at 68 for the magnetite-liquid system. In our MD simulations, the time step was set to 0.5 fs and configurations were stored every 1000 steps. The temperature was kept constant at 200 or 300 K, controlled with a Langevin thermostat applied only to four inner layers in the bulk of the magnetite slab. In this way, molecules at the interface are thermalized via interactions with surrounding molecules without any direct correction of their velocities, which could affect dissociation and recombination events. The volume was kept constant and the pressure fluctuated around 0 bar with a large variance. The committee of NNPs led the MD simulations with a committee disagreement threshold set to 2 eV/Å. During all our runs only a few configurations (order of 10) exhibited higher disagreement.

**Table 1:** Types and Features of the MD Simulations

	coverage [H_2_O/u.c.]	size [u.c. × u.c.]	*T* [K]	time [ns]
trimers dynamics	3	1 × 1	300	54
	0.75	2 × 2	300	34
				
water ground states	6	1 × 1	200	400
	6	1 × 1	300	400
	8	1 × 1	200	350
	8	1 × 1	300	350
				
dissociation mechanism and water MSD and diffusion	1	3 × 3	300	100
	4	3 × 3	300	100
	6	6 × 6	300	50
	8	6 × 6	300	50
				
bulk water	68	4 × 4	300	29

#### 2.3.2 Density Profiles and Octahedral-Tetrahedral Split

We computed the surface density profile of oxygen and hydrogen atoms of water on the SCV. As a reference point, we used the average *z*-coordinate, determined at each time step, of the iron and oxygen atoms in the outer surface layer, where the *z*-axis pointed normal to the surface. The distances of the water atoms from the surface were then binned into histograms. To get the density profiles, the histograms were normalized by dividing by the bin length, the number of time steps, and the number of unit cells so that the integral over a specific interval of distances from the surface gave the average number of atoms per unit cell that were in that interval of distances.

To refine the information provided by the density profiles, geometric details of the surface were introduced. The SCV features an alternating pattern of an outer row of octahedral irons and oxygens and an underlying row of tetrahedral irons (more precisely, irons that would have been octahedrally/tetrahedrally coordinated in the bulk), with one dissociation site per unit cell (see Figure 2). We exploited the chemical differences between these rows and subdivided the hydrogen and oxygen atoms of the water above them into two groups: if the *xy* projection of an atom was between the surface oxygen diagonals next to the octahedrally coordinated irons (see Figure 2), the atom was labeled as *octahedral* or *oct*; otherwise, it was labeled as *tetrahedral* or *tet*. The surface oxygen rows were determined at every time step by evaluating the oxygens’ *xy*-coordinates projected on the antidiagonal, which allowed us to group them and get for each row an average value.

## 3 Results

In this section, we present our findings obtained from MD simulations (Table 1) performed with the final NNPs committee. We begin with the analysis of the low water coverage on the magnetite surface, followed by the liquid water interface with magnetite.

### 3.1 Low Water Coverage on Magnetite

#### 3.1.1 Trimer Dynamics

**Figure 3:**
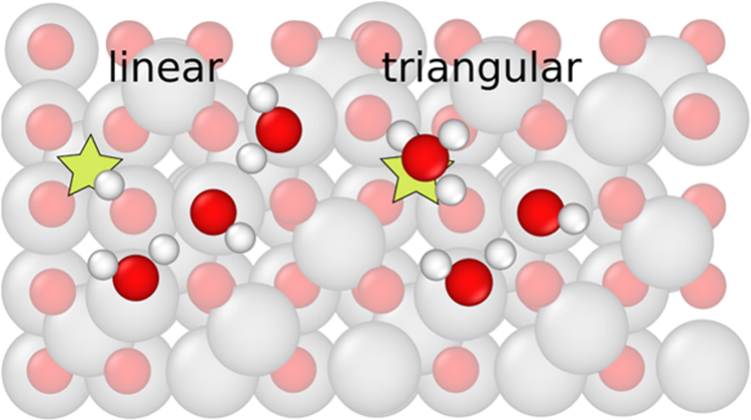
Linear and triangular water trimers on the SCV of magnetite. In both configurations, a water molecule dissociates, donating one hydrogen to the surface oxygen near the dissociation site (indicated by the star).

Experiments including atomic force microscopy and scanning tunneling microscopy in combination with DFT calculations have shown that partially dissociated water clusters of various sizes form at the SCV.^22^ One open question remains about the structure of the water trimer. In experiments, only linear trimers were observed. However, DFT calculations suggested that such linear trimers have the same adsorption energy as triangular trimers (Figure 3).

To address this issue, we have simulated three water molecules in contact with one SCV unit cell and with a replicated 2 × 2 unit cell (see details in Table 1). The starting water configurations were two types of trimers. These simulations reveal that the linear and triangular trimers exhibit distinct dynamics. To quantify this difference, we classified the water structures into clusters of different sizes at each time step, utilizing a sparse matrix to encode pair distances with a cutoff of 3.5 Å. By identifying clusters of three water molecules at the surface and distinguishing between the linear and triangular geometry, we calculated the probability of each trimer conformation. This probability was determined as the ratio of time spent in a particular conformation to the total time spent in any trimer form. While the results for the two independent surfaces of the same system agreed up to 0.01, the probability of being in a linear trimer was 0.72 for the one-unit cell system and 0.97 for the replicated one. This discrepancy indicated a large finite-size effect due to the interaction of the water cluster with its periodic image. Note also that the water coverage of the two systems was different (see Table 1). If we consider isolated trimers at the surface (well approximated by the replicated system) we can write
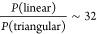
1That is, the linear trimer is more than 30 times more likely to occur than the triangular trimer at 300 K. This effect is likely entropic in origin because the potential energy of the two trimer configurations is essentially the same.

#### 3.1.2 Water Ground States

**Figure 4:**
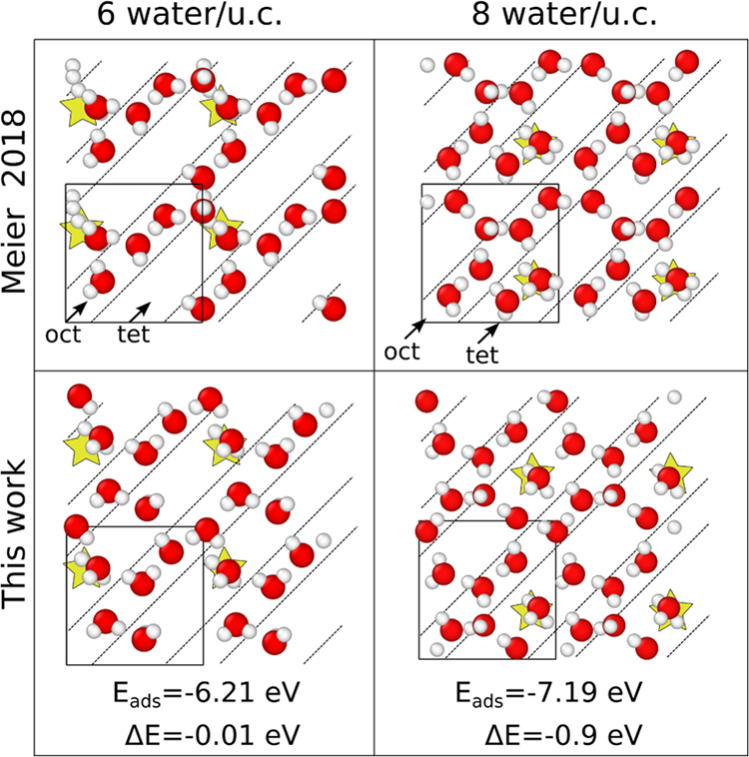
Minimum energy configurations of water on the SCV at low coverage. The configurations on the left involve six water molecules per unit cell, while on the right eight. The minima in the top row are from Meier et al.^22^ and the ones in the bottom row are new stable configurations (with Δ*E* < 0) discovered using the NNP. The SCV is not shown for a better visualization of the water structure but the iron rows are indicated by the dashed lines and the dissociation site by the stars. The number of dissociated hydrogens in the previous minima are two while the new ground states have only one dissociated hydrogen.

Stable structures of water on the SCV were found to preferentially arrange as shown at the top of Figure 4 for 6 and 8 water coverages. We ran in parallel different MD simulations (details in Table 1) starting from both random configurations and the stable structure reported in ref (22). During the MD runs, the systems with random initial structures relaxed, dissociating one water molecule, while the systems initiated with stable configurations eventually drifted away from the initial state.

We picked configurations equally spaced in time and produced from the two surfaces involved in the simulation, two independent water arrangements. The obtained structures were relaxed by minimizing the energy using the NNP. This procedure was computationally very efficient with the NNP, allowing a quick initial energy comparison of a large number of structures (on the order of tens of thousands). Then, only the 100 lowest energy structures were minimized via DFT. All of the candidates which showed lower energy than the already established configurations were selected, and their water structures were sliced out and placed on top of the exact same magnetite slab in order to isolate the water contribution. Finally, these setups were further minimized via DFT. Following this procedure, we found new configurations, shown in the bottom row in Figure 4 for 6 H_2_O/u.c. (left) and for 8 H_2_O/u.c. (right), with comparable and lower energies (Δ*E* = −0.01 and Δ*E* = −0.9 eV respectively) to the previously reported ones (top row). The new structures are different: while the previous minima have two dissociations per unit cell, the new ground states present only one dissociation per unit cell. The adsorption energy, defined as *E*_ads_(*n*) = *E*_tot_(*n*) – *E*_slab_ – *nE*_H_2_O_, with *n* being the number of water molecules in the system, was computed for the new minima
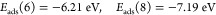


#### 3.1.3 Dissociation Mechanism

**Figure 5:**
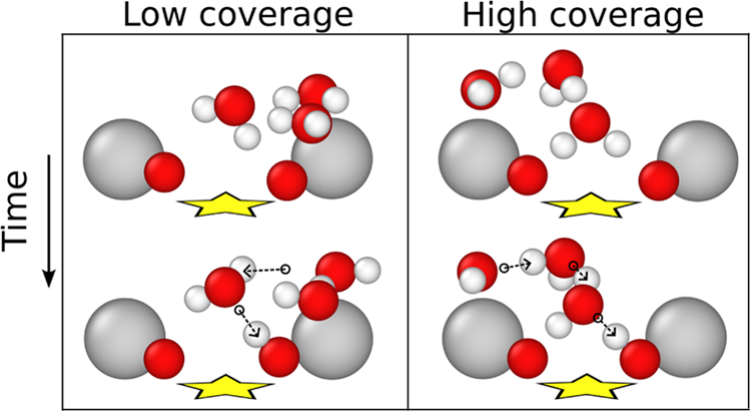
Snapshots of the dissociation mechanism from the simulations. The left and right panels present the dissociation with low and high amounts of water, respectively. The top and bottom figures are the configuration pre- and after-dissociation, respectively. Water molecules not involved in the process are omitted. The dashed arrows show proton transfers.

The mechanism of water dissociation has not been studied to date. We used the NNP to investigate such events, observing them closely in simulations (see Table 1). The process of water dissociation depends on the amount of water on the surface, but the initial requirement for a dissociation event, independent of the coverage, is that a water molecule must position itself directly above the dissociation site. At this stage, for water agglomerates of two or three molecules, one hydrogen of the water molecule points toward a surface oxygen forming a strong hydrogen bond with the surface (see the left panel in Figure 5). However, the molecule does not yet dissociate at this point. For that to happen, one or two water molecules must adsorb on a neighboring octahedral iron, forming a hydrogen bond with the initially adsorbed water molecule. Only then can a surface oxygen capture one hydrogen from the water molecule on top of the vacancy, initiating dissociation (we call this hydrogen atom the *dissociated hydrogen* in the following). Almost simultaneously (within a few femtoseconds), the water on the dissociation site in turn accepts one hydrogen atom from the neighboring water molecule. As a result, two hydroxides form. This process is the formation mechanism of the dimer and trimer configurations,^22^ with a dissociated hydrogen and an OH ion on the octahedral rows.

Increasing in coverage, beyond ∼3 H_2_O/u.c., the water molecules organize in larger clusters, and more than three water molecules can surround a dissociation site. In this situation, shown in the right panel of Figure 5, the water molecule on top of the dissociation site points its dipole vector toward the surface with symmetric hydrogen bonds to the surface oxygens and an extra water molecule bridges the proton transfer from this water molecule and the one adsorbed on the octahedral iron. The waiting time for a dissociation at high coverage is lower than at low coverage. In rare case, the dissociation follows an alternative mechanism, in which first a hydronium is formed which then donates a hydrogen to the surface oxygen.

The dissociation events have important consequences for the diffusion of water on the SCV, which we discuss in the following section.

#### 3.1.4 Water Mean Square Displacement and Diffusion

**Figure 6:**
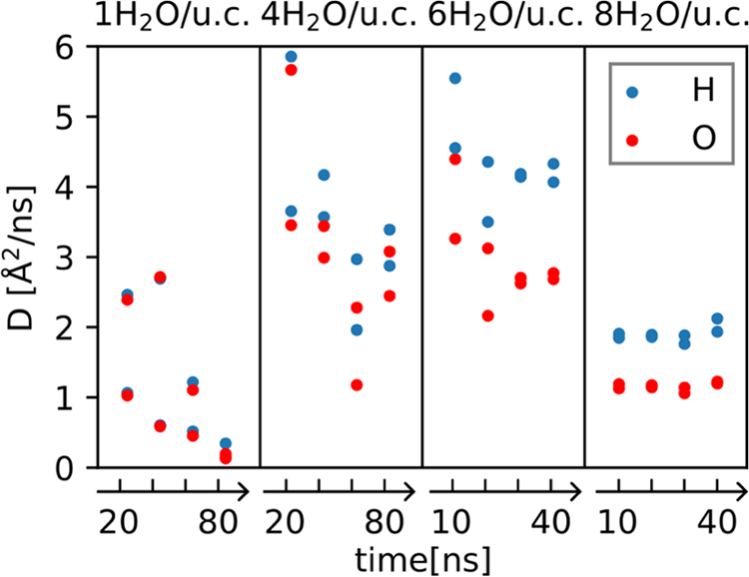
Diffusion coefficient *D* of oxygens (red) and hydrogens (blue) for a coverage of 1, 4, 6, and 8 H_2_O per unit cell. The diffusion coefficients are obtained from slices of the respective trajectories increasing in time after an initial equilibration period. The results are shown for both of the two independent interfaces present in the simulation setup.

The batch of trajectories of the previous section were extended beyond the dissociation times (see Table 1) in order to determine the two-dimensional average mean square displacements (MSD) of the oxygens and hydrogens of the water molecules at the SCV surface. After an equilibration time of 20 ns (10 ns) for 1 and 4 H_2_O/u.c. (6 and 8 H_2_O/u.c.), we have divided the rest of the trajectories into four equal parts of 20 ns (10 ns) time length and extracted four diffusion coefficients from linear fits to the MSDs. The results of this analysis are shown in Figure 6 for each of the coverages separately for O and H and the two independent surfaces.

The diffusion coefficients of hydrogen and oxygens of water in a system with 1 H_2_O/u.c. decrease with time due to the dissociation of water molecules: once water clusters stabilize by dissociating the hydrogen, water dimers and trimers are pinned near the dissociation site. As the recombination event is extremely rare at 300 K, these water molecules are essentially immobile for the entire simulation. At this stage, contributions to the mean square displacement come from the rest of the water molecules that roam detaching from one water cluster and attaching to another. The more water molecules a cluster has, the higher the probability that a water molecule at its border escapes. The pinned atoms are not the same during the simulations as water molecules can move eventually exchanging positions. Therefore, for water agglomerates, the kinetics of water molecules consists of a sequence of hops, in which particles are trapped for a while until some cooperative motion unlocks a path in the vicinity and allows a few molecules to jump away. This mechanism explains why the diffusion coefficient drops to a low value for 1 H_2_O/u.c systems. In the system with 4 H_2_O/u.c., the diffusion coefficient is very different for the two surfaces even after 100 ns, implying that the diffusion coefficient is affected by considerable statistical uncertainties in this case. Yet, we can make the qualitative observation that not only the number of dissociations is important but also where they occur at the surface. For 6 and 8 H_2_O/u.c., as the regime of approximately one dissociation per unit cell is reached, the diffusion coefficient converges for the two surfaces over time. Averaging the values of the diffusion coefficient of the last two runs (those converged) of 6 H_2_O/u.c. and all the 8 H_2_O/u.c. runs we obtained the following diffusion coefficients given in units of in [Å^2^/ns]
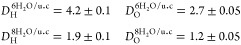
2

Notably, the diffusivity of the hydrogen atoms is larger than that of the oxygen atoms. The reason for this is again related to dissociation. The presence of OH^–^ ions in the water layer stimulates hydrogen exchanges, resulting in an overall higher mobility of the hydrogen atoms. Hence, hydrogen atoms have two ways to diffuse: one as a part of a water molecule and the other one as hydrogen transfer (H_2_O + OH^–^ → OH^–^ + H_2_O).

**Figure 7:**
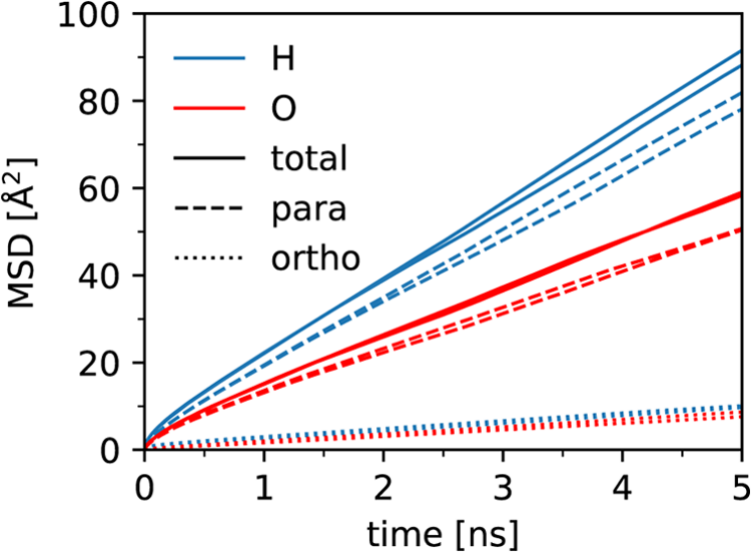
Two-dimensional MSD of water oxygens (red) and hydrogens (blue) at the magnetite SCV interface. The total, parallel, and orthogonal MSD (solid, dashed, and dotted lines, respectively) is shown for the 6 water molecules per unit cell system, for both the two surfaces of the slab.

We also computed the directional MSDs parallel and orthogonal to the iron rows for the converged systems, as shown in Figure 7. The ratio of parallel and perpendicular diffusion coefficients of the oxygen is
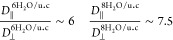
3Therefore the water molecules diffuse mainly along the iron rows, implying that the free energy barrier across octahedral-tetrahedral iron rows is greater than that between the octahedral iron.

### 3.2 Liquid Water Interface with Magnetite

**Figure 8:**
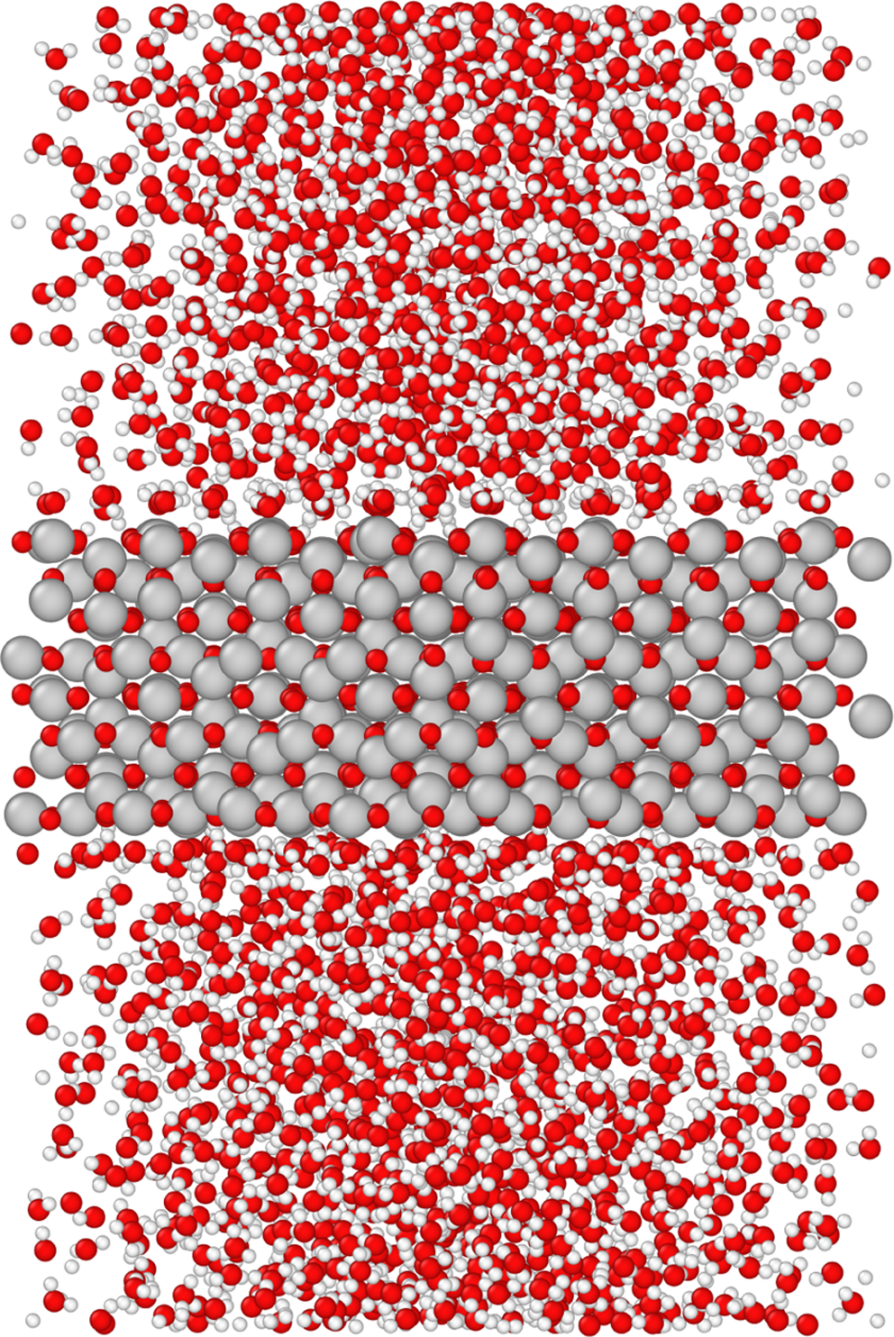
A snapshot of the simulation of the magnetite-liquid water interface. The top surface is oriented with the direction of the iron rows perpendicular to the plane of the figure. From this perspective, an order of the water near the surface is evident: from dissociated hydrogen at the surface to well-aligned water adsorbed on the octahedral rows.

We simulated the magnetite SCV in contact with a slab of liquid water of roughly 56 Å (2176 total water molecules). The setup is shown in Figure 8 and the details of the run are in Table 1. The system was equilibrated first at 0 bar using an NP_*z*_T simulation, in which the simulation box was free to adjust in the *z*-direction. Then, during the initial equilibration phase lasting about 10 ns, the water organizes and dissociates at the surface, eventually reaching a stationary regime. The analysis presented below focuses on the remaining 19 ns of the trajectory. We examine the statistical distribution of water configurations at the surface, averaging both over time and the two surfaces of the magnetite slab.

**Figure 9:**
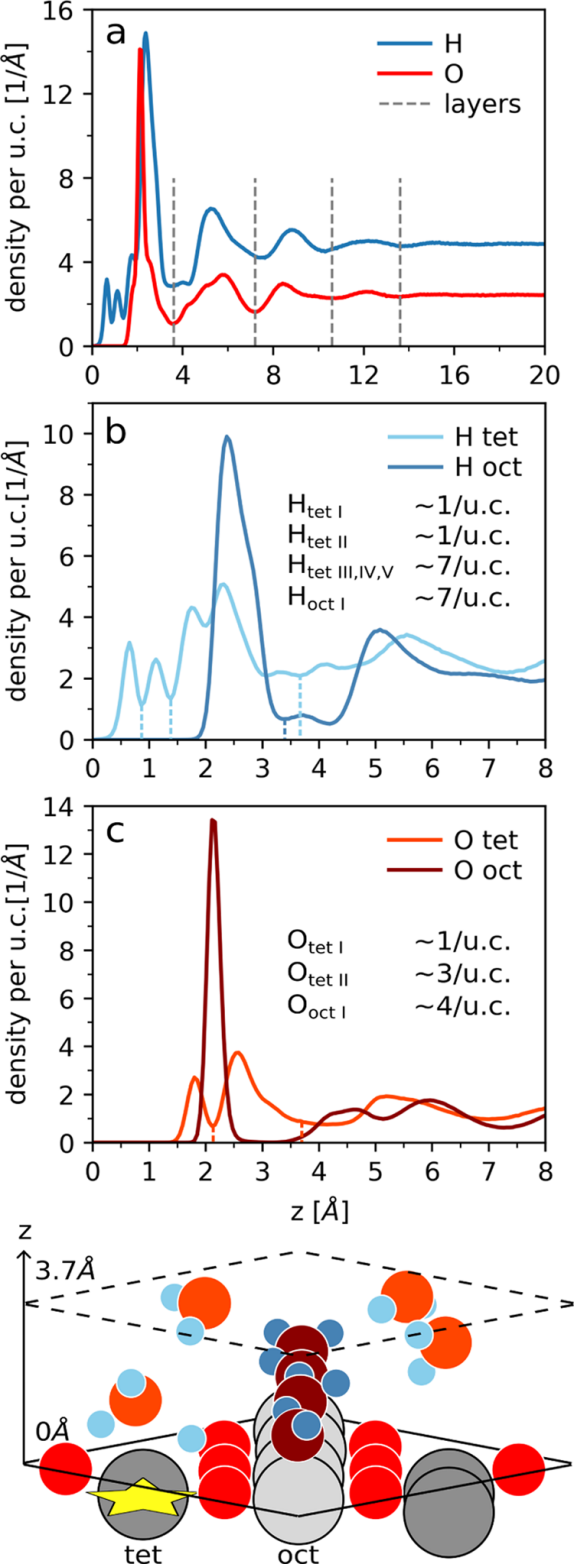
Plot (a) shows the density profile of H and O separately as a function of the distance *z* from the interface. The dashed gray lines indicate the minima in the O density, subdividing the water in layers. Plots (b, c) show the profile of H and O, respectively, but distinguished with colors based on their location on top of octahedral or tetrahedral iron rows. The result of the peak integration is also displayed, and the dashed lines delimit the integration regions. The sketch represents the structure of the water at the magnetite surface in the first layer. The same color legend of plots (b, c) is used.

In particular, we computed the density profile of oxygen and hydrogen atoms in the liquid water phase above the SCV (see Section 2.3 for details). As can be seen in Figure 9a, the water molecules are organized differently in terms of density than in isotropic bulk water up to around 15 Å from the interface. Focusing on the oxygen distribution, a distinct layering of water at the surface can be observed: four peaks and minima define four hydration layers located at heights of 3.6, 7.2, 10.6, and 13.8 Å. We find that the first two oxygen peaks consist of multiple subpeaks, indicating the presence of a finer layering structure.

In order to refine the density profile, we distinguish between oxygens and hydrogens at octahedral and tetrahedral sites (details in Section 2.3). The corresponding density profiles, shown in Figure 9b,c, yield a more detailed understanding of the structure of the first water layer (sketched at the bottom of Figure 9).

The first tetrahedral hydrogen (light blue) peak arises from dissociated hydrogens, which bend from their surface oxygen toward the dissociation site. The second peak is slightly wider but smaller, and it is attributable to hydrogens that are less constrained in their movements and are involved in a hydrogen bond with the surface. By integrating the first two peaks of Figure 9b, we expect the number of dissociated hydrogens per unit cell to be 0.94 while the number of H-bonded hydrogen accounts for 0.93 atoms. The water molecules on top of the dissociation sites donate H-bonds to the surface oxygens. The oxygens of these water molecules produce the first well-defined tetrahedral peak (orange in Figure 9c) that yields one atom if integrated up to 2.2 Å from the surface. These integrals of the peaks are all close to unity, indicating the stability of the configuration established between the dissociated hydrogen with the opposing surface oxygen and the water molecule on top of the dissociation site (as sketched in Figure 9).

The next peak to emerge corresponds to oxygens located along the octahedral iron rows (dark red in Figure 9c). The sharpness of the peak is a measure of the strength of the interaction of the water oxygen with the magnetite irons. The peak integrates four atoms: one water oxygen bonds to one octahedral iron, fully covering the row. The corresponding octahedral hydrogens (dark blue in Figure 9b) also produce a sharp peak with a shoulder on the right. This implies that the OH bonds of the water molecules on the octahedral sites are partially horizontal on the plane and partially pointing away from the surface. The average number of hydrogens in the octahedral region of the first layer is 7, as one hydrogen is dissociated at the surface, and the OH^–^ is delocalized within the chain of water molecules adsorbed onto the octahedral iron rows.

The oxygens above the tetrahedral irons (second orange peak in Figure 9c) complete the first layer. These are not anymore bonded to the surface as they produce a broad peak, which starts only at 2.2 Å and lacks a clear ending. Imposing an average of 3 tetrahedral oxygen atoms (one per tetrahedral iron), we infer that the first layer extends to about 3.7 Å. The hydrogens needed in the first layer in the tetrahedral region to balance the charge and complete 4 water molecules are 7.3 (recalling that the tetrahedral water on the dissociation site has 0.93 H/u.c. under the second H peak), which is the value obtained when integrating up to the fifth tetrahedral peak (from 1.4 to 3.7 Å).

The second layer is already less structured. Nevertheless, from the octahedral and tetrahedral splitting of the water density, we can roughly estimate the average number of molecules in this layer. The two octahedral peaks account for 4.2 O/u.c. (dark red in Figure 9c, from 3.5 to 7.2 Å) while the tetrahedral peak accounts for 4.1 O/u.c. (orange in Figure 9c from 3.6 to 7 Å), resulting in a total of ca. 8.3 O/u.c. in the second layer.

## 4 Discussion and Conclusions

In this work, we have developed an NNP of the Behler-Parrinello architecture for the magnetite/water interface. We have produced a DFT-based data set for the SCV terminated magnetite in contact with water molecules, which we cleaned of “corrupted” data encountered due to the instability of DFT calculations for this material. As the manual inspection was not sufficient to spot every error, we leveraged the prediction of the committee of NNPs to remove unsuitable configurations from the data set. Although this procedure may seem arbitrary, it proved very effective in the production of a cleaned data set. The training of the final NNP enabled for the first time stable and efficient simulations with *ab initio* accuracy of the SCV of magnetite in contact with water, otherwise impossible with direct DFT-MD. The new NNP can handle all the water coverages, from bare surfaces to fully covered slabs, across a wide temperature range up to 400 K (for a simpler system as one and two water per unit cell one can also safely run at 500 K). The potential is reactive, and the dissociation of water molecules at the surface can be simulated. These features were essential for conducting the simulations of the SCV in contact with water agglomerates and liquid water, which were used to produce all of our results.

The dynamics of two energetically equivalent water trimers could be distinguished from the analysis of the trajectory of the replicated 2 × 2 system. New ground states of water on the SCV were discovered after long NNP-MD runs. These structures need to be verified in the future with X-ray Photoelectron Spectroscopy (XPS) or Atomic Force Microscopy (AFM) experiments. The exploration of the energy minima for a larger system with more water molecules is also intriguing, as the experimental results indicate that for higher coverages the water ground state could break the one-unit cell periodicity.^22^ Our NNP-based simulations provided insights into the dissociation mechanisms, and we obtained qualitative and quantitative information on the diffusion of water molecules on the SCV. The extensive MD simulations of the liquid water/magnetite interface allowed us to gather enough statistics to determine a detailed density profile. Our results indicate that water organizes at the interface in four different layers visible in the oxygen’s density profile and recovers bulk-like behavior only after 15 Å. Incorporating geometrical information on the SCV into the density profiles, in particular by dividing the water environment depending on its location above tetrahedral or octahedral iron rows, enabled us to gain a comprehensive understanding of the structure of water in the first layer. Important properties of the magnetite/water interface, such as its vibrational spectroscopy and the dissociation kinetics of water molecules, are left for future study.

An issue with the developed NNP is its intrinsic locality. The long-range interactions that might produce an effect in the presence of ions are neglected. The inclusion of such forces into the potential and the study of their role in the dissociations are left for future studies. Another limitation lies in its restriction to water in the SCV model. This weakness hinders exploration of the magnetite exposure to water in other directions. Nevertheless, we are confident that, by augmenting the training set with structures featuring different terminations, our model could be generalized to encompass alternative surfaces (like the (111) termination^12,17,20^), potentially unveiling new stable surface structures and reconstructions.

## Data Availability

The training set, the architecture, and the weights of the NNPs of the committee used in this work are publicly available on Zenodo (https://zenodo.org/records/13810783). In the repository, the configurations of the newly discovered water minima can also be found.
